# LIGHT/TNFSF14 is increased in patients with type 2 diabetes mellitus and promotes islet cell dysfunction and endothelial cell inflammation in vitro

**DOI:** 10.1007/s00125-016-4036-y

**Published:** 2016-07-15

**Authors:** Bente Halvorsen, Francesca Santilli, Hanne Scholz, Afaf Sahraoui, Hanne L. Gulseth, Cecilie Wium, Stefano Lattanzio, Gloria Formoso, Patrizia Di Fulvio, Kari Otterdal, Kjetil Retterstøl, Kirsten B. Holven, Ida Gregersen, Benedicte Stavik, Vigdis Bjerkeli, Annika E. Michelsen, Thor Ueland, Rossella Liani, Giovanni Davi, Pål Aukrust

**Affiliations:** 1 Research Institute of Internal Medicine, Oslo University Hospital Rikshospitalet, Sognsvannsveien 20, 0372 Oslo, Norway; 2 K.G. Jebsen Inflammatory Research Center, University of Oslo, Oslo, Norway; 3 Institute of Clinical Medicine, Faculty of Medicine, University of Oslo, Oslo, Norway; 4 Center of Excellence on Aging, University of Chieti, Chieti, Italy; 5 Institute for Surgical Research, Oslo University Hospital Rikshospitalet, Oslo, Norway; 6 Section for Transplantation Surgery, Oslo University Hospital Rikshospitalet, Oslo, Norway; 7 Department of Endocrinology, Morbid Obesity and Preventive Medicine, Oslo University Hospital Rikshospitalet, Oslo, Norway; 8 Lipid Clinic, Oslo University Hospital Rikshospitalet, Oslo, Norway; 9 Department of Nutrition, Institute of Basic Medical Sciences, University of Oslo, Oslo, Norway; 10 National Advisory Unit on Familial Hypercholesterolemia, Oslo University Hospital Rikshospitalet, Oslo, Norway; 11 Section of Clinical Immunology and Infectious Diseases, Oslo University Hospital Rikshospitalet, Oslo, Norway; 12 K.G. Jebsen TREC, University of Tromsø, Tromsø, Norway

**Keywords:** Cytokines, Endothelial cells, Inflammation, Insulin, Islets, Type 2 diabetes

## Abstract

**Aims/hypothesis:**

Activation of inflammatory pathways is involved in the pathogenesis of type 2 diabetes mellitus. On the basis of its role in vascular inflammation and in metabolic disorders, we hypothesised that the TNF superfamily (TNFSF) member 14 (LIGHT/TNFSF14) could be involved in the pathogenesis of type 2 diabetes mellitus.

**Methods:**

Plasma levels of LIGHT were measured in two cohorts of type 2 diabetes mellitus patients (191 Italian and 40 Norwegian). Human pancreatic islet cells and arterial endothelial cells were used to explore regulation and relevant effects of LIGHT in vitro.

**Results:**

Our major findings were: (1) in both diabetic cohorts, plasma levels of LIGHT were significantly raised compared with sex- and age-matched healthy controls (*n* = 32); (2) enhanced release from activated platelets seems to be an important contributor to the raised LIGHT levels in type 2 diabetes mellitus; (3) in human pancreatic islet cells, inflammatory cytokines increased the release of LIGHT and upregulated mRNA and protein levels of the LIGHT receptors lymphotoxin β receptor (LTβR) and TNF receptor superfamily member 14 (HVEM/TNFRSF14); (4) in these cells, LIGHT attenuated the insulin release in response to high glucose at least partly via pro-apoptotic effects; and (5) in human arterial endothelial cells, glucose boosted inflammatory response to LIGHT, accompanied by an upregulation of mRNA levels of *HVEM* (also known as *TNFRSF14*) and *LTβR* (also known as *LTBR*).

**Conclusions/interpretation:**

Our findings show that patients with type 2 diabetes mellitus are characterised by increased plasma LIGHT levels. Our in vitro findings suggest that LIGHT may contribute to the progression of type 2 diabetes mellitus by attenuating insulin secretion in pancreatic islet cells and by contributing to vascular inflammation.

**Electronic supplementary material:**

The online version of this article (doi:10.1007/s00125-016-4036-y) contains peer-reviewed but unedited supplementary material, which is available to authorised users.

## Introduction

Type 2 diabetes mellitus is associated with accelerated atherogenesis, resulting in premature ischaemic manifestations of coronary, cerebrovascular and peripheral arterial disease, which contribute greatly to the increased morbidity and mortality in these patients [1, 2]. Inflammation seems to promote increased insulin resistance and impaired beta cell function in the pancreas, and inflammatory mediators also contribute to vascular pathology and accelerated atherogenesis in this disorder [1–3]. The identification of the relevant inflammatory mediators in these processes is, however, not fulfilled.

TNF superfamily (TNFSF) member 14 (LIGHT/TNFSF14) is a cytokine in the TNFSF [4], signalling through TNF receptor superfamily member 14 (HVEM/TNFRSF14) and the lymphotoxin β receptor (LTβR) [3, 4]. LIGHT is primarily expressed on T cells and dendritic cells, but has also been found in platelets, monocytes and granulocytes, being involved in innate and adaptive immunity as well as in the regulation of cell survival and proliferation [4, 5]. Studies in animal models and some clinical studies indicate that LIGHT may be crucial for the development of various inflammatory disorders [6, 7], and it has also been implicated in the pathogenesis of atherosclerosis and vascular inflammation [8, 9]. Recently, LIGHT has been shown to regulate lipid homeostasis [10] and has been associated with obesity, potentially through promotion of inflammatory responses in adipocytes [11, 12]. LIGHT has also been implicated in the immune-mediated beta cell destruction in diabetes [13], but data on the involvement of LIGHT in type 2 diabetes mellitus are scarce.

Based on its role in vascular inflammation and its recently discovered role in metabolic disorders, we hypothesised that LIGHT could be involved in the pathogenesis of type 2 diabetes mellitus. This hypothesis was investigated by various experimental approaches, including clinical studies in patients with type 2 diabetes mellitus and experimental studies in human pancreatic islet cells and human arterial endothelial cells (HAECs).

## Methods

### Participants

#### Italian cohort

A cohort of 191 patients (87 women, 104 men; mean age 65 ± 8 years) with type 2 diabetes mellitus were enrolled at the Diabetes Clinic of Chieti University Hospital from 2008 to 2011, as previously described [14] (Table 1). Exclusion criteria were: (1) clinically significant hepatic, renal, cardiac or pulmonary insufficiency; (2) history of malignant neoplasms (diagnosed and treated within the last 5 years); (3) autoimmune disorders and type 1 diabetes mellitus; (4) a recent history (<6 months) of thrombotic events, pregnancy or lactation; and (5) regular use of estroprogestin, iron, antioxidants, non-steroidal anti‐inflammatory drugs or antiplatelet agents other than aspirin (acetylsalicylic acid [ASA]). Of the diabetic patients, 94 were treated with low‐dose aspirin (100 mg/day) for the prevention of primary or secondary cardiovascular events. Diabetic patients with arterial hypertension or hypercholesterolaemia were included if well controlled with stable drug therapy: 108 (56.5%) had arterial hypertension and 134 (70.2%) were hypercholesterolaemic in accordance with Adult Treatment Panel (ATP) III criteria.

**Table 1:** Baseline characteristics of the Italian type 2 diabetic patients

Variables	Patients with type 2 diabetes			
	All (*n* = 191)	Not taking aspirin (*n* = 97)	Taking aspirin (*n* = 94)	*p* value^a^
Men, *n* (%)	104 (54.4)	47 (48.5)	57 (60.6)	0.11
Age, median (IQR), years	65 (60–70)	64 (59–70)	66.0 (61–69)	0.139
BMI (kg/m^2^)	28.1 (25.1–31.1)	28.7 (25.3–32)	28.1 (24.8–30.8)	0.191
Diabetes duration, years	5 (1–11.5)	1 (1–7.2)	7 (3–20)	<0.0001
Smoking	4 (2.1)	1 (1.0)	3 (3.2)	0.084
Diabetes duration > 1 year, *n* (%)	111 (58.1)	39 (40.2)	72 (76.6)	<0.0001
Systolic BP (mmHg)	135 (125–140)	130.5 (120–145)	135 (130–140)	0.354
Diastolic BP (mmHg)	80 (70–85)	80 (76–90)	80 (70–82)	0.017
Fasting plasma glucose (mmol/l)	7.44 (6.55–8.5)	7.72 (6.77–9.51)	7.11 (6.16–8.27)	0.006
HbA_1c_ (mmol/mol)	52 (46–58)	52 (46–56)	52 (46–61)	0.603
HbA_1c_ (%)	6.9 (6.4–7.5)	6.9 (6.4–7.3)	6.9 (6.4–7.7)	0.603
Hypertension, *n* (%)	108 (56.5)	48 (49.5)	60 (63.8)	0.002
Hypercholesterolaemia, *n* (%)	134 (70.2)	63 (64.9)	71 (75.5)	0.112
Total cholesterol (mmol/l)	4.93 (4.31–5.64)	4.96 (4.49–5.68)	4.78 (4.00–5.45)	0.038
HDL-cholesterol (mmol/l)	1.25 (1.04–1.49)	1.31 (1.11–1.52)	1.21 (0.98–1.45)	0.086
Triacylglycerols (mmol/l)	1.39 (0.96–1.93)	1.50 (1.00–2.09)	1.34 (0.90–1.89)	0.233
LDL-cholesterol (mmol/l)	2.88 (2.31–3.46)	2.96 (2.43–3.49)	2.72 (2.17–3.43)	0.125
Microvascular complications, *n* (%)	28 (14.7)	8 (8.2)	20 (21.3)	0.002
Macrovascular complications, *n* (%)	38 (19.9)	3 (3.1)	35 (37.2)	<0.0001
Previous MI, *n* (%)	7 (3.7)	0 (0)	7 (7.4)	0.001
Previous stroke, *n* (%)	3 (1.6)	0 (0)	3 (3.2)	0.052
Previous TIA, *n* (%)	5 (2.6)	0 (0)	5 (5.3)	0.006
Carotid stenosis, *n* (%)	6 (3.1)	0 (0)	6 (6.4)	0.006
Medical treatment				
Statin, *n* (%)	52 (27.2)	15 (15.5)	37 (39.4)	<0.0001
Metformin, *n* (%)	73 (38.2)	28 (28.9)	45 (47.9)	0.007
PPAR-γ, *n* (%)	11 (5.8)	1 (1.03)	10 (10.6)	0.009
Sulfonylurea, *n* (%)	38 (19.9)	13 (13.4)	25 (26.6)	0.036
Insulin, *n* (%)	18 (9.4)	4 (4.1)	14 (14.9)	0.021
Glinide, *n* (%)	8 (4.2)	0 (0)	8 (8.5)	0.006
Incretin, *n* (%)	0 (0)	0 (0)	0	–
Ezetimibe, *n* (%)	1 (0.5)	1 (1.0)	0 (0)	1.000
Fibrate, *n* (%)	4 (2.1)	0 (0)	4 (4.3)	0.057
PUFA, *n* (%)	8 (4.2)	2 (2.1)	6 (6.4)	0.167
ACE inhibitor, *n* (%)	47 (24.6)	23 (23.7)	24 (25.5)	0.862
ARB, *n* (%)	31 (16.2)	10 (10.3)	21 (22.3)	0.027
Diuretic, *n* (%)	32 (16.8)	13 (13.4)	19 (20.2)	0.237
β-blocker, *n* (%)	22 (11.5)	5 (5.2)	17 (18.1)	0.006
CCA, *n* (%)	23 (12.0)	11 (11.3)	12 (12.8)	0.824
PPI, *n* (%)	21 (11)	4 (4.1)	17 (18.1)	0.016

^a^By Mann–Whitney, *χ* ^2^ or Fisher’s exact test, as appropriate

ACE, angiotensin-converting enzyme; ARB, angiotensin receptor blocker; CCA, calcium channel blocker; IQR, interquartile range; PPAR-γ, peroxisome proliferator-activated receptor γ; PUFA, polyunsaturated fatty acids; MI, myocardial infarction; PPI, proton pump inhibitor; TIA, transient ischaemic attack

#### Norwegian cohort

A cohort of 40 Norwegian participants > 18 years of age (27 men, mean age 58 years) with type 2 diabetes mellitus were enrolled, regardless of type of glucose-lowering treatment, at the Diabetes Research Laboratory, Oslo University Hospital, from 2010 to 2012 (Table 2). Exclusion criteria included HbA_1c_ > 11% (97 mmol/mol), BMI > 45 kg/m^2^, malignancy, history of kidney stones, cardiovascular disease during the last 6 months, GFR < 30 ml min^−1^ 1.73 m^−2^, BP > 160/100 mmHg and chronic inflammatory disease in the active phase [15]. Seventeen patients (42.5%) were treated with low‐dose aspirin for the prevention of primary or secondary cardiovascular events. Patients with arterial hypertension (*n* = 35 [87.5%]) or hypercholesterolaemia (*n* = 34 [85.0%]) according to the ATP III criteria were included if well controlled with stable drug therapy.

**Table 2:** Baseline characteristics of the Norwegian type 2 diabetic patients (*n* = 40)

Variable	Median (IQR) or *n* (%)
Men, *n* (%)	27 (67.5)
Age (years)	58 (50–65)
BMI (kg/m^2^)	32.7 (28.9–36.6)
Diabetes duration, years	9 (3–15)
Smoking *n* (%)	10 (25.0)
Diabetes duration > 1 year, *n* (%)	39 (97.5)
Systolic BP (mmHg)	126 (121–137)
Diastolic BP (mmHg)	83 (80–92)
Fasting plasma glucose (mmol/l)^a^	8.86 (7.29–11.80)
HbA_1c_ (%)	7.3 (6.6–8.1)
HbA_1c_ (mmol/mol)	56 (49–65)
Hypertension, *n* (%)	35 (87.5)
Hypercholesterolaemia, *n* (%)	34 (85.0)
Total cholesterol (mmol/l)	4.10 (3.43–4.73)
HDL-cholesterol (mmol/l)	1.03 (0.82–1.20)
Triacylglycerols (mmol/l)	1.30 (0.90–1.80)
LDL-cholesterol (mmol/l)	2.30 (1.80–2.85)
Microvascular complications, *n* (%)^b^	6 (15.0)
Macrovascular complications, *n* (%)^b^	5 (12.5)
Medical treatment	
Statin, *n* (%)	27 (67.5)
Metformin, *n* (%)	27 (67.5)
PPAR-γ, *n* (%)	0 (0)
Sulfonylurea, *n* (%)	7 (17.5)
Insulin, *n* (%)	14 (35)
Glinide, *n* (%)	0 (0)
Incretin, *n* (%)	7 (17.5)
Ezetimibe, *n* (%)	0 (0)
Fibrate, *n* (%)	0 (0)
BP-lowering agent, *n* (%)	29 (72.9)
Aspirin, *n* (%)	17 (42.5)

^a^All patients had stopped oral glucose-lowering treatment 48 h before test and insulin 24 h before test

^b^Self-reported complications

IQR, interquartile range; PPAR-γ, peroxisome proliferator-activated receptor γ

#### Diabetes diagnosis

In both cohorts the diagnosis of type 2 diabetes mellitus was based on the American Diabetes Association guidelines [16]. Type 1 diabetes mellitus was suspected and excluded with islet autoantibody evaluation (i.e. GAD autoantibodies, islet antigen 2 [IA-2] antibodies, insulin autoantibodies) when one of the following applied: family history of type 1 diabetes mellitus, age < 40 years, lean phenotype or precocious requirement for insulin therapy. In the Norwegian cohort, anti-GAD and anti-IA-2 were measured in all participants. No patients were diagnosed clinically as having maturity onset diabetes of the young.

#### Control

The LIGHT levels in both cohorts were compared with LIGHT levels in 32 sex- and age-matched healthy Norwegian individuals (mean age 64 ± 5 years, 17 men and 15 women), based on disease history and clinical evaluation.

#### Consent

Written informed consent was obtained from each individual participating in the studies. The local ethics committees approved the protocols in both Italy and Norway.

#### Biochemical measurements

See the electronic supplementary material (ESM) Methods for details of biochemical measurements carried out in both cohorts.

### Human islet isolation

Human islets were isolated using a modified semi-automated digestion method [17] from nine male and female brain-dead donors aged 35–65 years provided by the islet isolation facility of the Nordic Network in Uppsala, Sweden, or at the islet isolation facility Oslo University Hospital in Norway after appropriate consent was given for multi-organ donation. Islet purity ranged between 70% and 90% as judged by dithizone staining, but the islet preparations were disqualified for clinical transplantation because of quantitative insufficiency. The cells were handpicked ensuring morphologically similar islets and exclusion of exocrine tissue. Descriptions of human islet culture and glucose-stimulated insulin secretion (GSIS) in human islet cells are given in the ESM Methods.

### Human arterial endothelial cell culture and stimulation

See ESM Methods for details.

### Isolation of peripheral blood mononuclear cells

Human peripheral blood mononuclear cells (PBMCs) were isolated from heparinised blood from seven patients with type 2 diabetes and six healthy controls by Isopaque Ficoll (Lymphoprep; Nycomed, Oslo, Norway) gradient centrifugation. Methodological details are given in the ESM Methods.

### Real-time quantitative RT-PCR

See the ESM Methods for details.

### Western blot

In human islets, western blotting was performed for protein analysis of LTβR, HVEM and glyceraldehyde 3-phosphate dehydrogenase (GAPDH). Methodological details are given in the ESM methods.

### Determination of beta cell death

Islet cell death was analysed by detection of DNA-histone complexes in the cytoplasmic fraction of cell lysates using a Cell Death Detection enzyme immunoassay (EIA) kit (Roche Diagnostics, Mannheim, Germany). See the ESM Methods for details.

### Preparation and culturing of platelet-rich plasma (PRP)

Preparation of citrated PRP was performed as described in the ESM Methods.

### EIAs

Levels of LIGHT in plasma, PBMC supernatants and PRP, and levels of IL-8 and monocyte chemoattractant protein 1 (MCP-1/CCL2) in HAEC supernatants, were measured by EIAs from R&D Systems (Abingdon, UK).

### Statistical methods

Differences in LIGHT levels were compared with the Mann–Whitney *U* test. If more than two groups were compared, the Kruskal–Wallis test was used a priori. Associations between LIGHT levels and clinical variables were analysed by Spearman’s rank correlation test or linear regression on log-transformed measures as necessary (normality assessed by the Kolmogorov–Smirnov test) prior to inclusion in stepwise regression. Data from in vitro studies were compared using the Mann–Whitney *U* test or Student’s *t* test as appropriate or Wilcoxon signed rank test for paired analysis. The *p* values are two-sided and considered significant when <0.05.

## Results

### Increased plasma levels of LIGHT in patients with type 2 diabetes mellitus

**Fig. 1:**
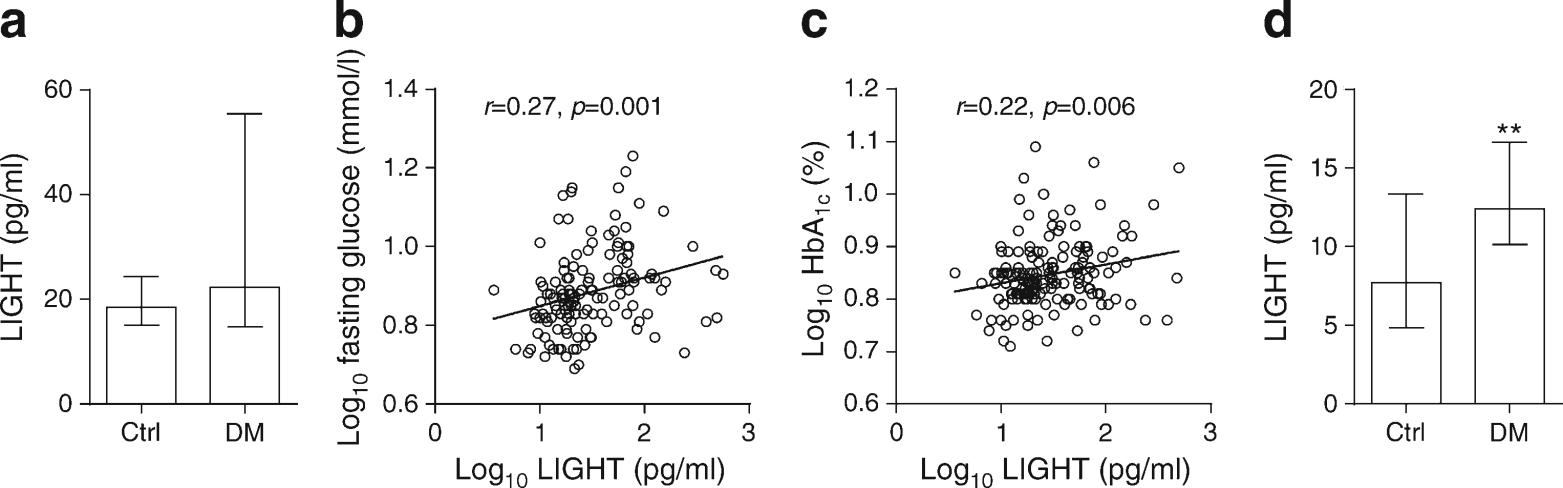
Circulating LIGHT levels in type 2 diabetes mellitus. (**a**) Plasma levels of LIGHT in 191 Italian type 2 diabetes patients and 32 healthy controls. (**b**, **c**) Correlations between plasma LIGHT levels and fasting glucose (**b**) and HbA_1c_ (**c**). (**d**) Plasma levels of LIGHT in 40 Norwegian type 2 diabetes mellitus patients and 32 healthy controls. Correlations are given as Pearsons *r* between log_10_-transformed values while box plots represent median and 25th and 75th percentiles. The Mann–Whitney *U* test was used to compare patients and controls. **p < 0.01 vs controls. Ctrl, controls; DM, diabetic group

Plasma levels of LIGHT were significantly raised in 191 patients with type 2 diabetes mellitus (Italian cohort) compared with 32 age- and sex-matched healthy controls (Fig. 1a). Within the Italian cohort, LIGHT was significantly correlated with glycaemic control as assessed by fasting plasma glucose (*r* = 0.27, *p* = 0.001; Fig. 1b) and HbA_1c_ levels (*r* = 0.22, *p* < 0.006; Fig. 1c). When the patients were stratified according to time since diagnosis (≤1 year [*n* = 42], 2–9 years [*n* = 43] and ≥ 10 years [*n* = 40]), there was a gradual increase in LIGHT levels according to disease duration (ESM Fig. 1). However, whereas these three groups are comparable for most of the clinical characteristics, they were different in relation to age, ongoing aspirin treatment and glucose-lowering medication (ESM Table 1), weakening the impact of the association between LIGHT and disease duration.

We aimed to replicate our findings in another type 2 diabetes mellitus cohort by measuring plasma LIGHT levels in 40 patients with this disorder recruited at Oslo University Hospital. As in the Italian cohort, the Norwegian cohort had significantly raised plasma levels of LIGHT compared with the 32 age- and sex-matched healthy controls (Fig. 1d). We were, however, not able to confirm the association between LIGHT and disease duration (*r* = −0.23, *p* = 0.18), fasting glucose (*r* = 0.09, *p* = 0.58) or HbA_1c_ (*r* = 0.27, *p* = 0.18) in the Norwegian population, potentially reflecting that the Italian population was larger (*n* = 191) and phenotypically different. Indeed, only one out of 40 of the patients in the Norwegian cohort had disease duration < 1 year compared with 80 out of 191 in the Italian cohort. Moreover, the Norwegian population had higher BMI, longer disease duration and higher HbA_1c_ and fasting glucose (Tables 1 and 2). It is possible that the correlation with LIGHT and glycaemic control is attenuated in more advanced disease.

### Platelets from type 2 diabetes mellitus spontaneously increase LIGHT release

**Fig. 2:**
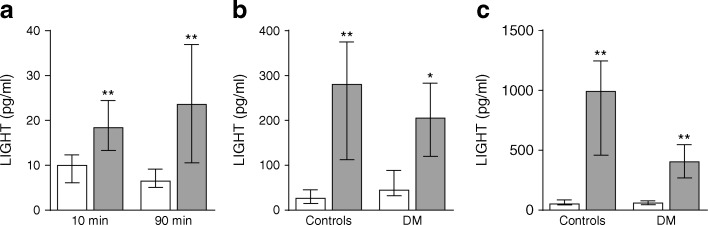
Release of LIGHT from platelets and PBMCs. (**a**) The spontaneous release of LIGHT in PRP from seven patients with type 2 diabetes mellitus (grey bars, Norwegian cohort) and six healthy controls (white bars). (**b**, **c**) The release of LIGHT in unstimulated PBMCs (white bars) and PBMCs stimulated with phytohaemagglutinin (PHA; 20 μg/ml, grey bars) after culturing for 20 h (**b**) and 48 h (**c**) in the same individuals as in (**a**). The Mann–Whitney *U* test was used to compare patients and healthy controls (**a**) and Wilcoxon signed rank test to compare PHA-stimulated and unstimulated cells (**b, c**). *p < 0.05 and **p < 0.01. DM, diabetic group

Platelets are known as a cellular source of LIGHT in plasma [5] and, as shown in Fig. 2a, platelets (i.e. PRP) from patients with type 2 diabetes mellitus (*n* = 7) spontaneously released a significantly higher amount of LIGHT than platelets from healthy controls (*n* = 6) after both 10 and 90 min incubations. Based on plasma concentrations (∼15–25 pg/ml), these data suggest that platelets are an important cellular source of circulating LIGHT levels in our type 2 diabetes mellitus cohorts. In the Italian cohort, we previously measured urinary 11-dehydro-TXB_2_ excretion rate and soluble CD40 ligand (sCD40L) as markers of platelet activation [14]; both variables were correlated with plasma levels of LIGHT (*r* = 0.035, *p* = 0.055 and *r* = 0.277, *p* = 0.001, respectively), further supporting a link between platelet activation and circulating LIGHT levels. Somewhat surprisingly, however, there was no difference in LIGHT level between those who were treated with aspirin and those who were not in either the Italian cohort (median [25th–75th percentile]: 21.8 [15.3–43.7] pg/ml vs 24.1 [14.4–56.7] pg/ml, *p* = 0.91, aspirin users [*n* = 94] and non-users [97], respectively) or the Norwegian cohort (11.7 [10.0–19.2] pg/ml vs 12.6 [11.0–16.3] pg/ml, aspirin users [*n* = 17] and non-users [*n* = 23], respectively).

### Activated PBMCs release large amount of LIGHT

Activated T cells and monocytes are important cellular sources of LIGHT [7] and, as shown in Fig. 2b, phytohaemagglutinin (PHA)-activated PBMCs released a large amount of LIGHT with the same pattern in type 2 diabetes mellitus patients (*n* = 7) and controls (*n* = 6). Whereas these results are not relevant for the circulating LIGHT levels in our diabetes cohorts, they could be relevant to the release of LIGHT from infiltrating T cells and monocytes within the vessel wall and pancreas in type 2 diabetes mellitus patients.

### LIGHT and its receptors are upregulated by inflammatory stimuli in human pancreatic islet cells

**Fig. 3:**
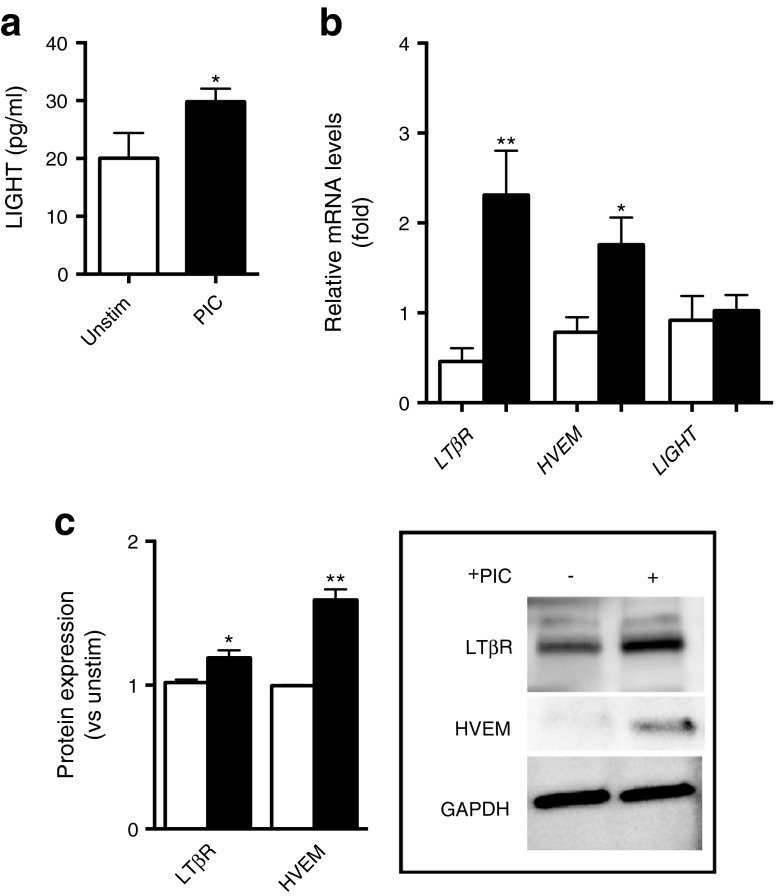
Proinflammatory stimuli increase the expression of LIGHT in human islets. Islets from independent preparations were stimulated for 24 h (**a**, **b**) or 48 h (**c**) with (black bars) or without (white bars) a PIC (IL-1β [1 ng/ml], IFN-γ [50 ng/ml], and TNF [10 ng/ml]) before the levels of LIGHT (ng/ml) were determined by ELISA in cell supernatant fractions (**a**). (**b**) Expression of the LIGHT receptors (*LTβR* and *HVEM*) and *LIGHT* mRNA levels as assessed by quantitative PCR in relation to the control gene β actin. (**c**) Protein levels of the LIGHT receptors in relation to the protein expression levels of GAPDH as assessed by western blotting. Data are presented as mean ± SEM (*n* = 3–6). *p < 0.05 and **p < 0.01 vs unstimulated cells using a Mann–Whitney *U* test. Unstim, unstimulated

To further elucidate the association of LIGHT with type 2 diabetes mellitus, we examined the regulation and effects of LIGHT in human pancreatic islet cells. Inflammation, and in particular IL-1β, has been implicated in the pathogenesis of beta cell dysfunction in type 2 diabetes mellitus [3, 18]. Therefore, the cells were co-stimulated with a proinflammatory cytokine cocktail (PIC) of IL-1β (10 ng/ml), TNF (10 ng/ml) and IFN-γ (50 ng/ml) (see ESM Methods). This mixture of inflammatory stimuli significantly enhanced the release of LIGHT into cell supernatant fractions and markedly upregulated the mRNA levels of the two LIGHT receptors *HVEM* (also known as *TNFRSF14*) and *LTβR* (also known as *LTBR*) without any significant effect on *LIGHT* (also known as *TNFSF14*) mRNA levels after culturing for 24 h (Fig. 3a, b). This increase in *HVEM* and *LTβR* expression in PIC-exposed islets was also seen at the protein level, as determined by western blotting (Fig. 3c).

### LIGHT reduces insulin release and increases cell death in human islets

**Fig. 4:**
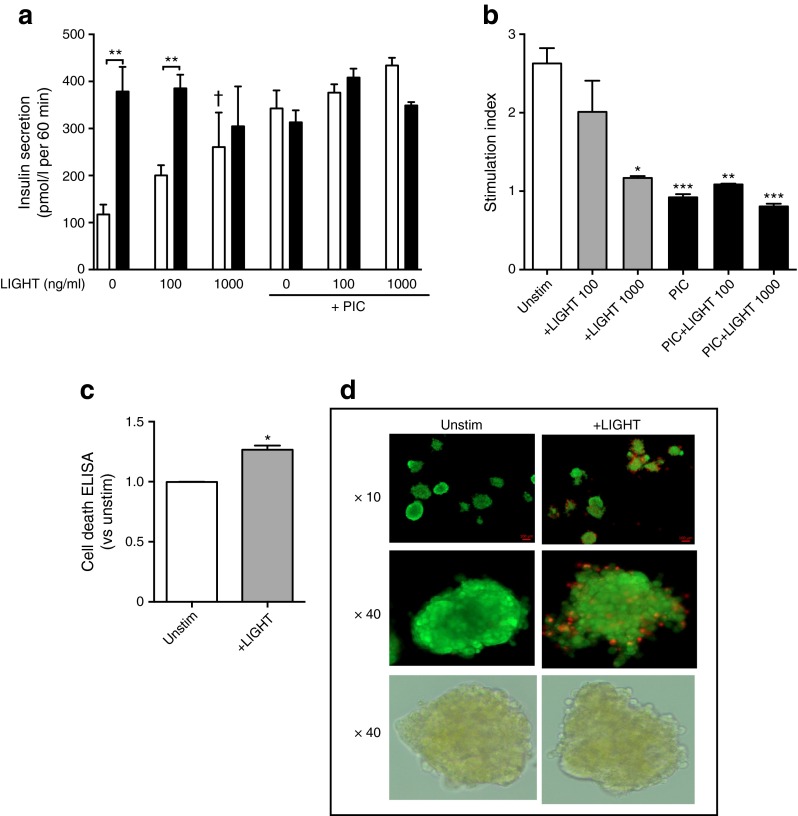
LIGHT decreases insulin secretion and viability in human islets. Islet potency, determined by insulin secretion, was measured by the GSIS test performed in human islets cultured with LIGHT (100 and 1000 ng/ml), PIC (IL-1β [1 ng/ml], IFN-γ [50 ng/ml] and TNF [10 ng/ml]) or a combination thereof. GSIS was evaluated by 1 h incubation at 1.67 mmol/l (white bars), followed by 1 h incubation at 20 mmol/l glucose (black bars). Insulin secretion was measured in the respective supernatant fractions by ELISA (**a**) and calculated as the stimulation index (**b**) as detailed in ESM Methods. Cell death in LIGHT-exposed cells (1000 ng/ml, 48 h) was measured by Cell Death ELISA (**c**) and viability by fluorescent membrane integrity assay with fluorescein diacetate/propidium iodide (FDA/PI) staining of the same islets visualised by fluorescence microscopy, with bright-field images of the islets shown in the bottom row (**d**). Data are presented as mean ± SEM (*n* = 3–9). *p < 0.05, **p < 0.01 and ***p < 0.001 vs low glucose (**a**) or unstimulated cells (**b**); ^†^ *p* < 0.05 vs low glucose without LIGHT. All comparisons were made using the Mann–Whitney *U* test. Unstim, unstimulated

To elucidate the potential functional consequences of increased *LIGHT/HVEM/LTβR* expression in pancreatic islet cells, we examined the effects of LIGHT on insulin secretion in response to low (1.67 mmol/l) and high (20 mmol/l) glucose exposure. Whereas LIGHT increased insulin secretion in response to low glucose levels in a dose-dependent manner, LIGHT markedly inhibited insulin secretion in response to high glucose concentration (Fig. 4a, left). In fact, whereas high glucose levels induced a marked increase in insulin secretion compared with low glucose levels in unstimulated cells, the difference between high and low glucose exposure was nearly absent in LIGHT-stimulated (1000 ng/ml) cells. PIC induced a similar pattern as LIGHT, with minor differences in insulin release when comparing low and high glucose exposure with no additional effect of LIGHT (Fig. 4a, right). These patterns were also seen when the response was calculated as insulin stimulation index (i.e. the ratio of stimulated [high glucose exposure] to basal [low glucose exposure] insulin secretion) (Fig. 4b), with the suppressive effects of LIGHT being in the same order as those of the inflammatory cytokine cocktail (∼60% reduction). LIGHT has been shown to trigger apoptosis in various tumour cells [19] and, indeed, LIGHT-exposed islet cells show enhanced apoptosis as assessed by Cell Death ELISA (Fig. 4c) as well as enhanced propidium iodide staining (Fig. 4d).

### Glucose enhanced the inflammatory effects of LIGHT in HAECs

**Fig. 5:**
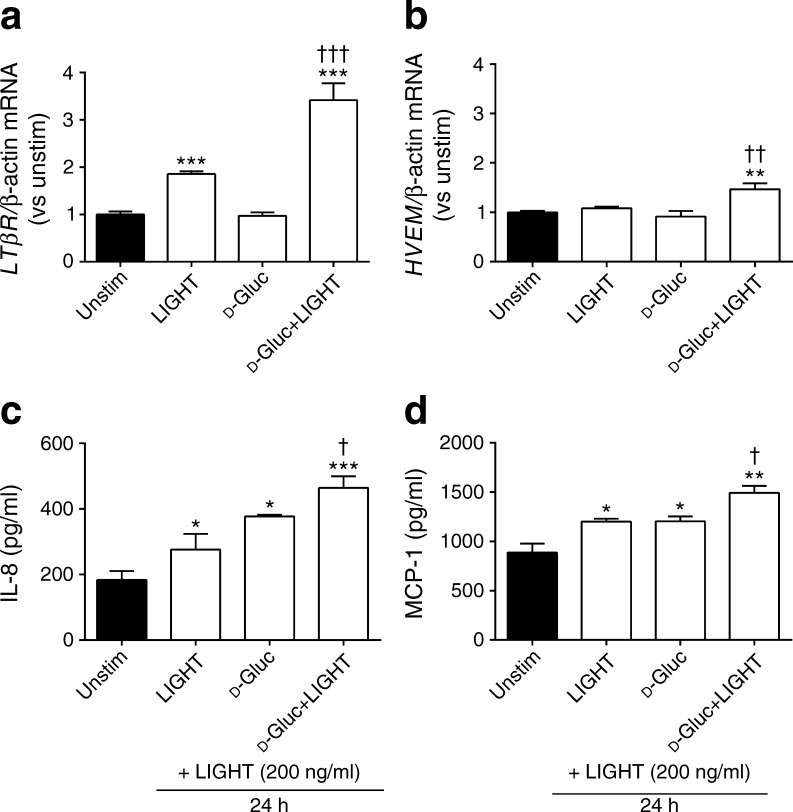
LIGHT increases the inflammatory potential of glucose-stimulated HAEC cells. HAECs were stimulated for 3 h (**a**, **b**) or 6 h (**c**, **d**) with either LIGHT (200 ng/ml), d-glucose (10 mmol/l) or a combination thereof. Gene expression of the *LTβR* (**a**) and *HVEM* (**b**) were examined by qPCR and data are given in relation to the control gene β-actin. The levels of IL-8 (**c**) and MCP-1 (**d**) were assessed in cell supernatant fractions by ELISA. (**c**, **d**) The cells were pretreated for 6 h as described above, followed by incubation with LIGHT (200 ng/ml) for 24 h. The medium was changed before the last incubation with LIGHT for 24 h. In all experiments, unstimulated cells received vehicle. Data are presented as mean ± SEM (*n* = 4–6). ^*^ *p* < 0.05, ^**^ *p* < 0.01 and ^***^ *p* < 0.001 vs unstimulated cells (Student’s *t* test). ^†^ *p* < 0.05, ^††^ *p* < 0.01 and ^†††^ *p* < 0.001 vs glucose or LIGHT alone. Gluc, glucose; Unstim, unstimulated

Vascular inflammation is an important complication of type 2 diabetes mellitus [2], and we and others have shown that LIGHT activates inflammatory responses in endothelial cells [5, 9, 20]. We therefore next examined the regulation of the two LIGHT receptors and the effect of LIGHT on inflammatory responses in HAECs with and without glucose exposure. First, LIGHT, especially when combined with glucose (10 mmol/l), markedly enhanced the expression of *LTβR* mRNA (Fig. 5a). Second, while glucose (10 mmol/l) and LIGHT (200 ng/ml) had no effect on *HVEM* expression, the combination of these stimuli induced a modest but significant effect on *HVEM* mRNA levels after culturing for 3 h (Fig. 5b). Third, pre-incubation of HAECs with glucose (10 mmol/l) and LIGHT (200 ng/ml) for 6 h resulted in the release of significantly higher levels of IL-8 and MCP-1, two prototypical endothelial-derived inflammatory chemokines, when the cells were further stimulated with LIGHT for an additional 24 h compared with cells pre-incubated with glucose or LIGHT alone (Fig. 5c, d). This finding suggests that glucose could enhance the LIGHT-mediated inflammatory response in arterial endothelial cells, potentially via a mechanism involving upregulation of its receptors in these cells.

## Discussion

**Fig. 6:**
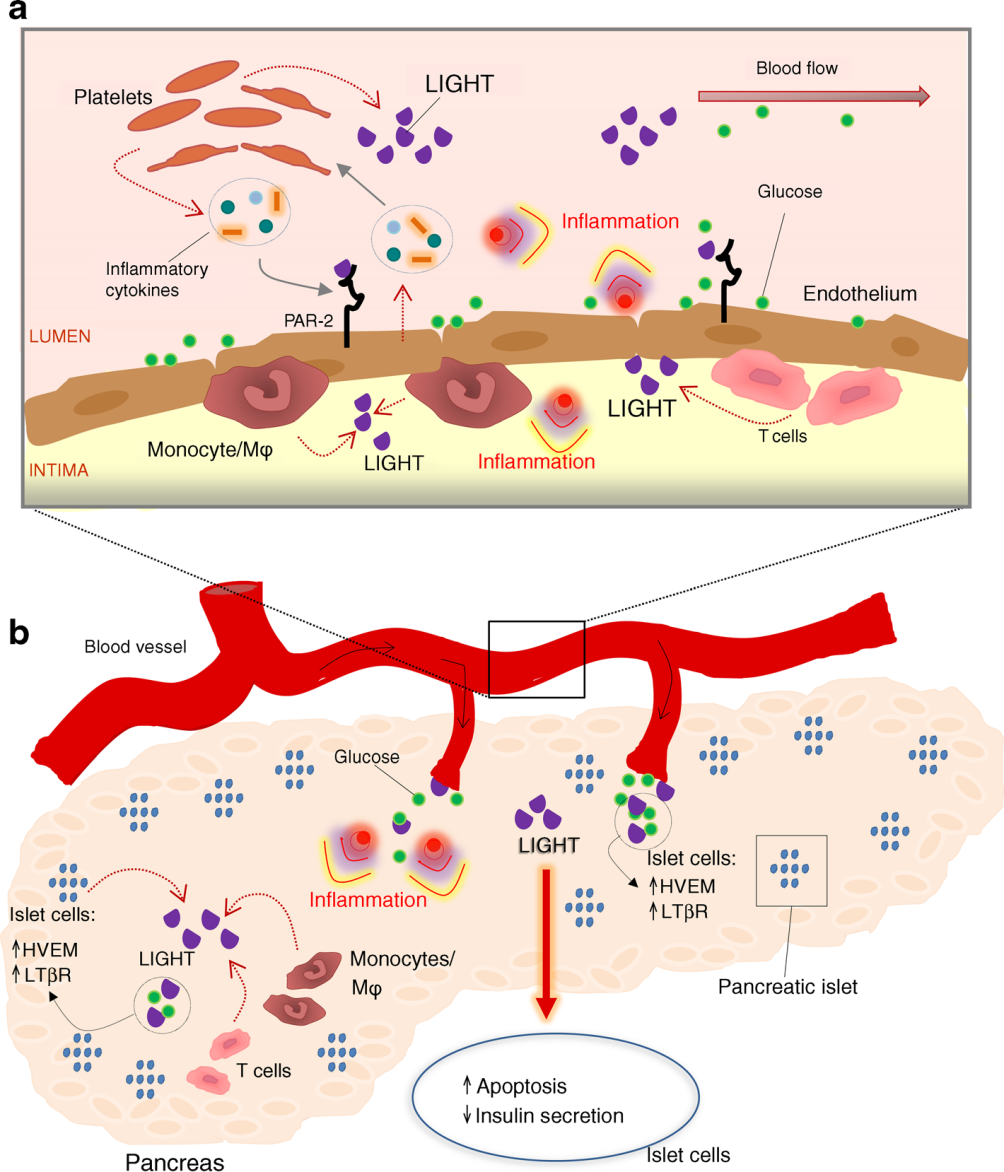
Hypothetical bidirectional interaction between glucose and LIGHT in type 2 diabetes mellitus. (**a**) The pancreas is heavily vascularised because of its function as sensor of blood glucose. In the circulation, platelets release LIGHT, as well as other cytokines, which exerts its effects through its receptors, HVEM and LTβR, on the endothelium, causing vascular inflammation. High glucose and increased PAR-2 expression increase the potency of LIGHT. When activated, the endothelium recruits T cells and monocytes/macrophages that release a large amount of LIGHT. (**b**) On inflammatory stimulation, pancreatic islets produce LIGHT accompanied with increased production of HVEM and LTβR. Recruited T cells and monocytes/macrophages also contribute to increased LIGHT levels. During high glucose exposure, LIGHT attenuates insulin release involving LIGHT-induced apoptosis of pancreatic islet cells, further contributing to hyperglycaemia. Thus, LIGHT could be part of a vicious circle leading to progression of type 2 diabetes mellitus. Mϕ, macrophage

In the present study we show that, as confirmed in two independent cohorts, the TNFSF member LIGHT is significantly increased in type 2 diabetes mellitus, potentially reflecting enhanced release from platelets in these patients. The LIGHT receptors HVEM and LTβR were found to be upregulated in pancreatic islet cells by inflammatory cytokines, and LIGHT itself attenuated the insulin release from these cells when exposed to high glucose levels via a mechanism that, at least partly, involved LIGHT-induced apoptosis of pancreatic islet cells. Finally, glucose boosted the inflammatory response of LIGHT in arterial endothelial cells, potentially through upregulation of the LIGHT receptors. Our findings suggest that LIGHT could be involved in the development and progression of type 2 diabetes mellitus and its complications, including the development of vascular inflammation via an inflammatory loop between platelets, endothelial cells, mononuclear blood cells and pancreatic islet cells that has LIGHT as an important link (Fig. 6).

LIGHT is involved in both innate and adaptive immune responses [4], and has been linked to various inflammatory and autoimmune disorders [6, 7, 21] Additionally, LIGHT has been shown to increase the uptake of modified lipids in macrophages [22], and induce hypertriglycerolaemia through inhibition of hepatic lipoprotein lipase [10]. LIGHT has also been linked to obesity in experimental models [11], contributing to metabolic-induced inflammation [12]. In experimental mice models, LIGHT has been associated with pancreatic islet cell apoptosis [13] and the development of diabetes through mediation of recruitment and activation of T cells into the islets [23]. Soluble LTβR has been shown to reverse spontaneous autoimmune insulitis in non-obese diabetic mice [24]. However, data on LIGHT in human diabetic disorders are scarce. Dandona et al reported raised LIGHT levels in a small cohort of 38 obese patients with no relation to type 2 diabetes mellitus [25]. Herein, we show increased LIGHT levels in individuals with type 2 diabetes mellitus compared with healthy controls as confirmed in two independent cohorts of 191 and 40 type 2 diabetes mellitus patients. Moreover, we show that soluble LIGHT and its receptors, HVEM and LTβR, are upregulated in pancreatic islet cells when the cells are exposed to inflammatory cytokines. Our findings further underscore a link between inflammation and type 2 diabetes mellitus, and show that LIGHT could be added to the list of mediators in the pathogenic loop between hyperglycaemia and inflammation in this disorder.

LIGHT is strongly expressed by activated T cells [4], and herein we show that activated PBMCs release a large amount of LIGHT. Several LIGHT-associated effects seem to be mediated by its membrane-bound form, in particular on T cells [10, 26]. Indeed, experimental studies on LIGHT and pancreatic islet cell pathology have focused on the membrane-bound LIGHT on T cells [23, 24]. However, Han et al showed that recombinant LIGHT could induce islet cell apoptosis in an experimental mouse model of islet cell transplantation [13]. Here, we show that soluble LIGHT can also affect the function of human islet cells. Thus, soluble LIGHT impaired insulin release when these cells were exposed to high glucose levels, potentially involving LIGHT-induced depletion of insulin when these cells are exposed to low levels of glucose as well as LIGHT-induced apoptosis of pancreatic islet cells. Thus, we can speculate that increased LIGHT levels in the pancreatic environment may alter the glucose dependence of insulin secretion, with excessive and unrequired insulin secretion in response to low glucose concentration and attenuated response to high glucose concentration.

We have previously shown that platelet-derived LIGHT is a potent inducer of inflammatory responses in endothelial cells [5]. Herein, we show that platelets from type 2 diabetes mellitus patients spontaneously release higher levels of LIGHT than platelets from healthy controls, and were an important cellular source of plasma levels of LIGHT in our diabetic cohorts. While the amount of LIGHT release from platelets may seem low, we have previously shown that when combined with other inflammatory mediators that are released from activated platelets, platelet-derived LIGHT has a significant impact on endothelial cell activation [5]. Activated T cells and monocytes are important cellular sources of LIGHT and herein we show that PHA-activated PBMCs release a large amount of LIGHT. Whereas these results are not relevant for the circulating LIGHT levels, they could be relevant to the release of LIGHT from infiltrating T cells and monocytes within the vessel wall or pancreatic islet cells. Thus, based on our experiments, both activated platelets that adhere to the vascular endothelium and infiltrating mononuclear cells could contribute to LIGHT-mediated vascular and pancreatic islet cell inflammation in type 2 diabetes mellitus. We have previously shown that activation of protease-activated receptor 2 (PAR-2) enhances the LIGHT-induced inflammatory responses in endothelial cells [9]. Interestingly, experimental studies suggest that PAR-2 is upregulated and show enhanced stimulatory responses in endothelial cells in diabetic mice [27, 28]. If a similar regulation of PAR-2 is also seen in type 2 diabetes mellitus, it will further enhance the inflammatory effect of LIGHT in these patients.

The present study has some limitations. The correlation of LIGHT with glycaemic control was found only in the large Italian cohort. The lack of data on insulin secretion as a fraction of the total insulin amount (content in islet + media) is another limitation. Nonetheless, our findings show that type 2 diabetes mellitus patients are characterised by increased plasma levels of LIGHT, and our in vitro findings suggest that LIGHT could contribute to the progression of this disorder by attenuating insulin secretion in pancreatic islet cells and by contributing to vascular inflammation.

## Electronic supplementary material

Below is the link to the electronic supplementary material.

ESM (PDF 203 kb)

## Abbreviations

EIA: Enzyme immunoassay
GAPDH: Glyceraldehyde 3-phosphate dehydrogenase
GSIS: Glucose-stimulated insulin secretion
HAEC: Human arterial endothelial cell
HVEM/TNFRSF14: TNF receptor superfamily member 14
LIGHT/TNFSF14: TNF superfamily member 14
LTβR: Lymphotoxin β receptor
MCP-1: Monocyte chemoattractant protein 1
PAR-2: Protease-activated receptor 2
PBMC: Peripheral blood mononuclear cell
PHA: Phytohaemagglutinin
PIC: Proinflammatory cytokine cocktail
PRP: Platelet-rich plasma
TNFSF: TNF superfamily
