# Supplementary material for: LIGHT/TNFSF14 is increased in patients with type 2 diabetes mellitus and promotes islet cell dysfunction and endothelial cell inflammation in vitro
Source: Diabetologia. 2016 Jul 15;59(10):2134–44. doi: 10.1007/s00125-016-4036-y (PMC5016561; doi:10.1007/s00125-016-4036-y)
Supplement: Supplementary file 1 — (PDF 203 kb) [file 125_2016_4036_MOESM1_ESM.pdf]

Electronic supplemental material (ESM)

Halvorsen B et al. **LIGHT/TNFSF14 is increased in patients with type 2 diabetes mellitus and promotes islet cell dysfunction and endothelial cell inflammation *in vitro***

Methods

Biochemical Measurements

Fasting plasma glucose was measured according to the glucose oxidase method. The HbA_{1c} level was determined by automated high-performance liquid chromatography (HPLC). The homeostasis model assessment of insulin resistance (HOMA-IR) was performed as described by Matthews et al. [1]. Total cholesterol, triglyceride, and high-density lipoprotein cholesterol concentrations were measured as previously described [2]. Low-density lipoprotein cholesterol was calculated using the Friedwald formula.

Human islet culture

Within 2-5 days from isolation, equal aliquots of the islet preparations were placed in 55-mm-Petri dishes (Sterilin, Heger AS, Oslo, Norway) and cultured in CMRL 1066 (Invitrogen, NV Leek, the Netherlands) containing 2.5% ABO-compatible human serum, 1% penicillin/streptomycin, 10 mM Hepes and 1% L-glutamine (Gibco, Paisley, UK) at 37°C (5% CO_2) and stimulated with (1) different concentration of recombinant human LIGHT (R&D Systems, Minneapolis, MN); (2) a mixture of inflammatory cytokines interleukin 1β (IL-1β, [1 ng/ml], interferon-γ [IFN-γ, 50 ng/ml], and TNF [10 ng/ml]); all from R&D Systems), or (3) a combination thereof for 24 or 48 hours as indicated. Cell pellets and supernatants were harvested and stored at −80°C until further analyses.

Glucose-stimulated insulin secretion in human islet cells

For static insulin secretion in response to a glucose challenger, following the standard operating procedure of the NIH Clinical Islet Transplantation Consortium, 20 islets were handpicked, transferred into 12-transwell trays (Costar, Cambridge, MA), and pre-incubated in Krebs-Ringer bicarbonate buffer supplemented with 1.67 mmol/l glucose at 37°C (5% CO_2) for 30 minutes. Thereafter, the islets were incubated for 1 hour in 1.67 mmol/l glucose (basal), before additional incubation for 1 hour in 20.0 mmol/l glucose (stimulated). Secreted insulin in the media was measured by enzyme immunoassay (EIA; Mercodia AB, Uppsala, Sweden). The capacity for insulin release was expressed as stimulation index (SI), calculated as the ratio of stimulated to basal insulin secretion.

In these experiments the standard operating procedure of the NIH Clinical Islet Transplantation Consortium where a fixed number of handpicked islets from each experimental condition are transferred into transwell trays for the glucose challenges of low and high glucose solution, assuming that the same islets are exposed to the different glucose solution. In the end of experiments the islets are stained with dithizone for recognition of only using islets (not exocrine cells) in the assay.

Human arterial endothelial cell culture

The human primary coronary artery endothelial cells (HAEC) were grown in complete Endothelial Growth Medium made up of Endothelial Basal Medium-2 supplemented with EGM-2 MV SingleQuot Kit and 10% fetal bovine serum (Lonza, Viviere, Belgium). Cells were seeded in 24-wells plates (Costar) in complete growth medium and grown for one to two days until 80% confluences. In these experiments we wanted to see if LIGHT had any functional effect on these cells, and therefore, we chose to perform these experiments on cells with <100% confluence. Thereafter the cells were washed once in Opti-MEM® (Life Technology) before stimulation for 3 hours with D-glucose (10 mM), LIGHT (200 ng/ml) or

combination thereof. Controls received vehicle. In some sets of experiments, the cells were boosted with the stimuli for 6 hours before a 24 hours incubation with LIGHT (200 ng/ml) in fresh serum free medium. Cell supernatants and cell pellets were harvested at indicated time and stored at -80°C until analyses with EIA or RT-PCR, respectively.

Real-time quantitative RT-PCR

Total RNA was extracted from frozen islet and HAEC pellets using the RNeasy mini kit (Qiagen, Hilden, Germany) and cDNA was prepared using the High-Capacity cDNA Archive Kit following the manufacturer's protocol (Applied Biosystems, Foster City, CA). Quantitative PCR detection was performed using the 7900 system with SYBR Green Master Mix and sequence-specific PCR primers designed by Primer Express software, version 3.0 (Applied Biosystems): *LTβR*, 5′-CGTCCCGCTCGTCAGAAA-3′ and 5′-CATAGGCGCTCCGCTGAA-3′ and *HVEM*, 5′-TCTGAGCCTGAGGCATGGA-3′ and 5′-GAGATACAGCACCAGCCTCAAGA-3′. Results were normalized to the housekeeping gene *β-actin* and data was analyzed using the standard curve method.

Western blot

Human islets (200 handpicked) were lysed in RIPA buffer with protease inhibitor (Life Technologies AS, Oslo, Norway). Samples were centrifuged and purified using QIAshredder purification column (QIAGEN). Protein concentration was determined using BCA assay (Pierce, Rockford, IL). Western blot was performed using Bio-Rad Mini-PROTEAN gels and the Mini-PROTEAN Tetra cell, Mini Trans-blot module. The following primary antibodies were used for detection: Rabbit anti-human LTβR (1:1500, LifeSpan Biosciences, Seattle, WA), rabbit anti-human HVEM, (1:1000, Novusbio, Littleton, CO), goat anti-human GAPDH (1:1000 Santa Cruz Biotechnology, Santa Cruz, CA). HRP conjugated anti-rabbit or anti-goat was purchased from Santa Cruz Biotechnology (1:10000). Chemiluminescence was performed using Clarity Western ECL substrate (Bio-Rad, Hercules, CA). Images were

acquired using a chemiDGC touch imaging system and analyzed using Image Lab Software (Bio-Rad).

Determination of beta cell death

Islets cell death was analyzed by detection of DNA-histone complexes in the cytoplasmic fraction of cell lysates using Cell Death Detection EIA kit (Roche Diagnostics, Mannheim, Germany) according to protocol offered by manufacturers or by fluorescein diacetate (FDA)/propidium iodide (PI) staining which stain viable cells (green) and dead cells (red), respectively. Briefly, a small fraction of the islets were incubated for 15 minutes in PBS containing final concentration FDA (0.46 μM) and PI (14.34 μM) (Sigma Aldrich) before images were taken using the Axio Observer Inverted Microscope (Carl Ziess AS, Munich, Germany) operating by ZEN lite software.

Stimulation of peripheral blood mononuclear cells (PBMC)

Freshly isolated peripheral blood mononuclear cells (PBMCs) were incubated in flat-bottomed 24-well microtitre plates ($2x10^6$ml, Costar) with RPMI 1640 medium (PAA Laboratories, Pasching, Austria) supplemented with 10% heat inactivated human AB+ serum with and without phytohaemagglutinin (PHA, 20 μg/ml; Thermo Scientific, Waltham, MA). Cell-free supernatants were harvested after 20 and 48 hours and stored at -80°C. The doses of PHA and the incubation time were based on preceding dose-response experiments.

Preparation and culturing of platelet-rich plasma (PRP)

Preparation of citrated PRP was performed as previously described [3]. Briefly, PRP was incubated at 22°C with Tris-buffered saline (pH 7.4; 20 mM Tris and 150 mM NaCl) only. At different time points, aliquots were removed and centrifuged at 13,000*g* for 5 minutes to obtain a platelet-free plasma which was stored at -80°C until LIGHT measurements.

References

1. Matthews DR, Hosker JP, Rudenski AS, Naylor BA, Treacher DF, Turner RC (1985) Homeostasis model assessment: insulin resistance and beta-cell function from fasting plasma glucose and insulin concentrations in man. Diabetologia 28:412-419
2. Lattanzio S, Santilli F, Liani R, et al (2014) Circulating dickkopf-1 in diabetes mellitus: association with platelet activation and effects of improved metabolic control and low-dose aspirin. J Am Heart Ass 3:e001000
3. Otterdal K, Smith C, Oie E, et al (2006) Platelet-derived LIGHT induces inflammatory responses in endothelial cells and monocytes. Blood 108:928-935

Supplementary Table S1. Baseline characteristics of the Italian type 2 diabetic (T2DM) patients according to disease duration.

Variables	Patients with Type 2 Diabetes Mellitus			
**Diabetes Duration (yr)**	**≤1**	**2-9**	**≥10**	***P* value**
**Male gender, n**	24	21	23	0.664
**Age (years) Median (IQR)**	61 (58-68)	62 (58-66)	69 (62.2-74.7)	<0.0001
**BMI (Kg/m^2)**	27.5 (24.4-30.2)	28.9 (27.3-31.2)	26.1 (24.1-31.5)	0.10
**ASA**	6	25	28	<0.0001
**Smoking**	0	2	2	0.248
**Fasting plasma Glucose (mmol/l)**	6.89 (6.29-7.65)	7.25 (6.39-9.47)	8.11 (7.11-9.15)	0.005
**Hemoglobin A_{1c} (mmol/mol)**	50 (45-53)	54 (49-61)	54 (50-62)	<0.0001
**Hemoglobin A_{1c} (%)**	6.7 (6.3-7.0)	7.1 (6.6-7.7)	7.1 (6.7-7.8)	<0.0001
**Hypertension, n**	23	24	26	0.362
**Hypercholesterolemia, n**	36	26	31	0.010
**Total Cholesterol (mmol/l)**	4.98 (4.44-5.81)	4.98 (4.39-5.69)	4.96 (4.28-5.58)	0.662
**HDL Cholesterol (mmol/l)**	1.32 (1.13-1.54)	1.15 (0.97-1.48)	1.16 (1.01- 1.34)	0.103
**Triacylglycerides (mmol/l)**	1.31 (0.98-1.77)	1.69 (0.98-2.62)	1.32 (0.98-1.86)	0.253
**LDL cholesterol (mmol/l)**	3.10 (2.39-3.86)	2.87 (2.20-3.44)	2.94 (2.42-3.46)	0.378
**Previous MI, n**	0	2	4	0.069
**Previous Stroke, n**	2	0	1	0.403
**Previous TIA, n**	2	1	2	0.772
**Carotid stenosis, n**	1	3	1	0.370

<u>**Medical treatment**</u>				
**Statins, n**	14	9	12	0.641
**Metformin, n**	3	29	23	<0.0001
**PPAR-γ n**	1	4	2	0.341
**Sulphonylureas, n**	1	8	18	<0.0001
**Insulin, n**	0	3	10	<0.0001
**Glinides, n**	0	1	3	0.118
**Incretins, n**	0	0	0	-
**Ezetimibe, n**	1	0	0	0.369
**Fibrates n**	0	2	0	0.144
**PUFA, n**	1	3	3	0.528
**ACE-inhibitors, n**	11	15	14	0.579
**ARBs, n**	6	6	6	0.99
**Diuretics, n**	7	11	7	0.526
**β-blockers, n**	3	6	7	0.357
**CCA, n**	6	4	8	0.379
**PPI, n**	3	7	5	0.324

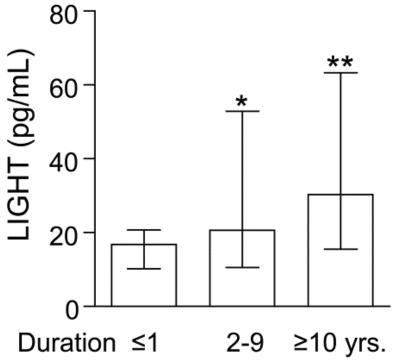

ESM Fig. 1. Plasma LIGHT levels plotted according to disease duration in the Italian cohort of type 2 diabetes mellitus (T2DM) patients (≤1 year [n=42], 2-9 years [n=43], ≥10 years [n=40]). Differences in LIGHT levels were compared with the Mann-Whitney U-test after the Kruskal-Wallis test was used *a priori*. *$p<0.05$ and **$p<0.01$ compared with ≤1 year duration.
